# A survey of accessibility and utilisation of chiropractic services for wheelchair-users in the United Kingdom: What are the issues?

**DOI:** 10.1186/2045-709X-19-20

**Published:** 2011-09-13

**Authors:** Naomi D McKay, Jennifer Langworthy

**Affiliations:** 1 Anglo-European College of Chiropractic, 13-15 Parkwood Road, Bournemouth, UK

**Keywords:** chiropractic, wheelchair-user, disabilities, complementary and alternative medicine, access

## Abstract

**Background:**

People with physical disabilities experience barriers to healthcare across all services despite a legal and moral obligation to the contrary. Complementary medicine is considered as supplementary to conventional care and integration of these approaches is essential to achieve optimal care. This paper explores the utilisation of chiropractic services and practitioner experiences of treating wheelchair-users which appears under-reported.

**Methods:**

A 20 item questionnaire was posted to 250 randomly selected chiropractors registered with the General Chiropractic Council. Follow-up questionnaires were sent 7 days after the initial return date. Quantitative data were subjected to frequency analysis.

**Results:**

The response rate was 64% (n = 161). The majority (66%) of chiropractors had been in practice less than 10 years and were practice owners (50%). Fifty-two percent of chiropractors sampled had treated a patient in a wheelchair in the previous 5 years. The majority (87%) had treated between 1 and 5 such patients. Patients with multiple sclerosis, stroke and cerebral palsy most commonly presented for treatment. The majority of patients' presenting complaint was musculoskeletal in origin, primarily for pain control. Only 13% of respondents worked in a fully accessible clinic. Impracticality of alterations was the most common reason for inaccessibility.

**Conclusions:**

Wheelchair-users seem to be an underserved patient group in relation to chiropractic services. Chiropractic management is primarily utilised for pain control in patients with physical disabilities in which mobility may be improved or maintained. Co-management of wheelchair-users with GPs appears to be desirable in order to achieve optimal patient care however more research is required regarding the efficacy of chiropractic treatment for a range of disabling conditions. Physical access was identified as a key barrier to accessing care.

## Background

The aim of all healthcare practitioners is to provide patients with the best possible service. However, in 2008, 32% of the 10.2 million people covered by the Disability Discrimination Act (DDA) experienced problems in the previous 12 months accessing pubic services, including healthcare [1,2]. There is a shortage of literature documenting the experiences of patients with physical disabilities when accessing healthcare however disabled patients have reported problems getting appointments at short notice and also at a time they have access to transport. In some cases patients have to battle the inaccurate assumption that their symptoms are somehow related to their disability [3]. Other barriers to healthcare include poor practitioner attitudes towards disability and limited physical access. These barriers have been linked to the development of preventable health complications [4].

Disability is defined by law [5] as "someone who has a physical or mental impairment that has a substantial and long-term adverse effect on his or her ability to carry out normal everyday activities." Although limited, evidence suggests that patients with physical disabilities consult complementary and alternative medicine (CAM) therapists including chiropractors, more often than the general population. Furthermore, they visited these practitioners more frequently, primarily for complaints of chronic pain and depression [6]. The majority of patients with physical disabilities appear to utilise CAM therapies that are manual rather than herbal in nature. This may be related to the symptoms commonly experienced by this patient group. Common therapies used by these patients include physiotherapy, aquatherapy, relaxation techniques, massage and chiropractic [6-8].

Evidence suggests that patients with disabilities consider complementary therapies as supplementary to, not a replacement for conventional care [7]. One study found that wheelchair-users with multiple sclerosis (MS) considered conventional care to be more beneficial than some CAM therapies for the treatment of their condition [9]. The integration of CAM with conventional medicine is however important as patients are entitled to full disclosure on all possible treatment options as a matter of autonomy [10].

While research exists which documents the experiences of wheelchair-users accessing CAM providers, the experiences of practitioners treating these patients appear under-reported. This study explored the utilisation of chiropractic services and potential barriers to care for wheelchair-users in the United Kingdom (UK) from the practitioner's perspective. Hitherto the term 'disability' is limited to someone who is reliant on a wheelchair for mobility.

## Methods

This study was internally reviewed by a review panel for ethics and feasibility. Following a ruling by the ethics committee that the study was sufficiently low risk, a 20 item questionnaire was constructed and subsequently piloted by 10 registered chiropractors. These data were excluded from final analysis. Using the random number generator 250 chiropractors were selected within SPSS version 17.0 for Windows XP. This was to represent 10% of the 2500 chiropractors registered with the General Chiropractic Council (GCC) in October 2009. Questionnaires were coded prior to distribution to enable follow-up of non-responders.

The questionnaire was posted to subjects with a cover letter and stamped addressed return envelope. Chiropractors who had treated a patient in a wheelchair since 2004 were eligible to answer the entire questionnaire. Those who had not could answer 9 out of the 20 questions. Participants were requested to return the questionnaire within 17 days of receipt. A second questionnaire was sent to non-respondents one week after the initial deadline, allowing a further 14 days for return. Participants were assured of confidentiality. Chiropractors were able to decline participation in the study by non-return of the questionnaire.

Data were collated using SPSS version 17.0 for Windows XP and were subjected to frequency analysis. Data were not used if a question had not been answered, if respondents had selected multiple responses inappropriately or where only one practitioner had reported a feature which did not appear in the choices provided. As such, none of the qualitative data gained was included in this study.

## Results

### Demographic

One-hundred and sixty-one of 250 chiropractors returned the questionnaire, providing a response rate of 64%. This represented only 6% of chiropractors registered with the GCC at the time of the study therefore the results cannot be assumed to be representative of chiropractors throughout the UK. Despite this, the issues identified in this paper may well exist beyond the sample studied. In this study 96 (60%) chiropractors sampled worked more than 37.5 hours per week. Eighty (50%) were practice owners, 56 (35%) associates, 6 (4%) partners in the practice and 18 (11%) rented a treatment room in another establishment. Fifty-eight (36%) of the chiropractors had been in practice for 5 years or less, 48 (30%) 6-10 years and 54 (34%) for 11 years or more. All results below are reported from the practitioner's perspective.

### Treatment numbers, presenting complaints and challenges

Approximately half (n = 83, 52%) of chiropractors sampled had treated a patient in a wheelchair in the previous 5 years. The majority (n = 72, 87%) had treated between 1 and 5 such patients, 9 (11%) between 6 and 10, while 2 (2%) had treated more than 10. Forty-three (52%) chiropractors reported the patient's general practitioner (GP) had been informed the patient was receiving chiropractic care. Table 1 identifies the specific disabling conditions and Table 2 details the primary presenting complaints of the wheelchair-users seen by the responding chiropractors.

**Table 1:** Most common disabling conditions in wheelchair-users encountered by chiropractors sampled (not exclusive)

Condition	n (%)
Multiple sclerosis	44 (53%)
Stroke	26 (31%)
Paralysis	24 (29%)
Cerebral palsy	23 (28%)
Trauma	20 (24%)
Congenital deformity	8 (10%)
Spina bifida	6 (7%)

**Table 2:** Most common presenting complaints in wheelchair-users encountered by chiropractors sampled (not exclusive)

Presenting complaint	n (%)
Neck pain	45 (54%)
Low back pain	41 (49%)
Other muscular pain	25 (30%)
Headache	10 (12%)
Depression	6 (7%)
Insomnia	5 (6%)

Table 2 Most common presenting complaints in wheelchair-users encountered by chiropractors sampled

Fifty-one (61%) chiropractors experienced challenges related to the management and treatment of this patient group and these are shown in Table 3. It is not known however whether these issues prevented or restricted the treatment of the patient.

**Table 3:** Specific challenges faced by chiropractors in the treatment of wheelchair-users (not exclusive)

Motivation	n (%)
Modifying treatment due to restriction of patient mobility	24 (29%)
Manoeuvring patients on and off treatment bench	20 (24%)
Accessing clinic and treatment room	8 (10%)
Managing increased physical load and manual handling	6 (7%)
Fitting treatment into standard appointment time	4 (5%)

### Motivation for treatment and key expectations

From the practitioner's perspective, the main motivation for wheelchair-users seeking chiropractic treatment is the patient's desire to utilise multiple therapies to manage their condition. The majority of patients sought care for pain control however the source of pain was not identified in this study. The main motivations for wheelchair-users seeking care and their key expectations of treatment are outlined in Tables 4 and 5 respectively.

**Table 4:** Practitioner-perceived motivations of wheelchair-users to seek chiropractic treatment (not exclusive)

Motivation	n (%)
Desire/need for multiple therapies for effective treatment	48 (58%)
Dissatisfaction with conventional care	30 (36%)
Trying something new/experimental	30 (36%)
Expectation of a chiropractic 'cure' or improvement	13 (16%)

**Table 5:** Key expectations for treatment of wheelchair-users seeking chiropractic care (not exclusive)

Key expectation	n (%)
Pain control	60 (72%)
Maintenance/improvement of mobility	36 (43%)
Relief of symptoms associated with their condition	35 (42%)
Relief of symptoms associated with wheelchair use	24 (29%)
Resolution of muscular problems	17 (20%)

### Access to Chiropractic Care

**Figure 1:**
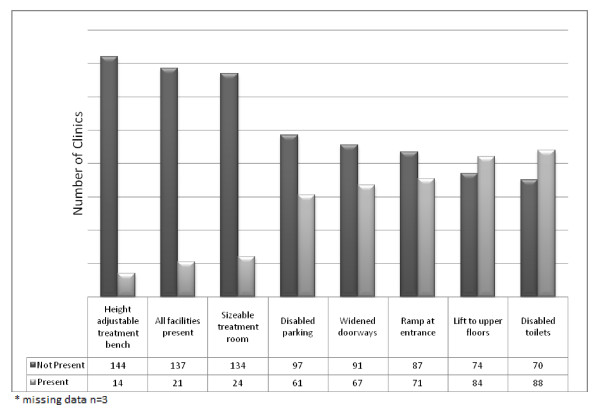
**Availability of Wheelchair Friendly Facilities in Chiropractic Clinic**.

Since being in practise, 8 (5%) respondents had refused to treat a wheelchair-user due to lack of access. A wheelchair user had never presented to 48 (30%) chiropractors surveyed at any point in their career. The availability of facilities for wheelchair-users in the sampled chiropractic clinics is outlined in Figure 1.

Factors that chiropractors identified which may prevent the adaptation of their clinic to increase disabled access are given in Table 6.

**Table 6:** Factors preventing the adaptation of chiropractic clinics to increase disabled access (not exclusive)

Factor	n (%)
Impracticality of alterations	86 (56%)
Limited funds	55 (36%)
Would not increase patient numbers	39 (25%)
Unaware of need to change	13 (8%)
Lack of time to make changes	11 (7%)

## Discussion

### Treatment of wheelchair-users

This study found that wheelchair-users are not commonly treated by the chiropractors sampled. In addition, the likelihood of treating such a patient does not appear to increase with time in practice.

The most common conditions encountered by chiropractors sampled were those in which mobility can be maintained or improved such as in MS and cerebral palsy. The presenting complaints encountered by chiropractors were primarily musculoskeletal in nature, which concurs with the findings of Rose [11] who found 38% of chiropractors sampled believed wheelchair-users require more treatment for musculoskeletal disorders. While more research is required regarding the efficacy of chiropractic treatment in wheelchair-users common sense dictates that a wheelchair user with normal anatomy receiving treatment has the potential to experience the same benefits as the general population. In addition, wheelchair-users may utilise chiropractic services more than the general population as although the health needs in disabled and able-bodied people are mostly the same, disabled patients are more prone to secondary conditions giving them a greater need of additional services [3].

In the current study, pain control was the most common reason patients in wheelchairs sought chiropractic treatment. Chiropractic care has already been found to be an effective treatment for chronic pain syndrome in MS [12]. This suggests chiropractic management may be effective in managing pain in other disabling conditions. Carson et al [8] found that 42% (n = 97) of 231 people with MS sampled used chiropractic care regularly. Of these, 81% (n = 79) use chiropractic to manage the symptoms of the disease. This is almost double the number of chiropractors who had treated a wheelchair-user for symptoms associated with their condition in the current study. However, the apparent high use of chiropractic services among MS patients compared to those with other disabling conditions may be misleading as the majority of research concerns these patients.

In the current study, 24 (29%) chiropractors had treated a wheelchair-user to relieve symptoms associated with their wheelchair use. Sitting for long periods in a confined space puts these patients at risk of developing muscle imbalances, fatigue and contraction, trigger points and pain [13]. Chiropractic has been shown to be effective in the treatment of myofascial complaints [14]. This may therefore be an area in which chiropractors can apply evidence-based treatment methods to wheelchair-users. The perceived key expectations of treatment of wheelchair-users in the current study included a combination of the factors listed as respondents were invited to select more than one option. This is important in the interpretation of results in which expectations may appear misleadingly distinct. In addition, it would have been useful for chiropractors to rate the importance of each expected treatment outcome in relation to each individual patient they had treated in order to increase accuracy of results. This however was beyond the scope of this study.

### Treatment Challenges

Fifty-one (61%) chiropractors surveyed experienced challenges specific to the treatment of wheelchair-users. According to the GCC, chiropractors should consider how services can be provided to anyone who may want to use them, including giving extra help for disabled users [15]. In order to achieve optimal care, practitioners are generally required to allow additional time and to have basic understanding of the nature of their patient's disability. Shinto et al [9] reported CAM therapists spend significantly more time with patients during treatment visits compared to their National Health Service (NHS) counterparts. While allowing extra time presented a challenge to a small minority of chiropractors in the current study, no chiropractor identified it as a reason to refuse treatment.

Manoeuvring a patient on and off the treatment bench presented a challenge for a substantial proportion of the chiropractors in the current study. Manual handling tasks are physically demanding, often unpredictable in nature and in the chiropractic setting, take place in an unfavourable environment [16]. However, while practitioner and patient safety is paramount, chiropractors must look to overcome these barriers. Of the chiropractors sampled, over one-quarter claimed to have modified their treatment in order to accommodate a wheelchair-user. Limited mobility does not therefore appear to be a factor which would prevent the treatment of wheelchair-users in chiropractic clinics. Indeed according to the GCC to do so would be unethical.

### The Role of Chiropractic Services

It is important to define the role of chiropractic services in the treatment of wheelchair-users. Iezzoni et al [17] concluded that people with disabilities were generally satisfied with conventional care and any dissatisfaction was largely related to quality of care in terms of physical access and practitioner knowledge regarding their condition. This would appear to be borne out by the view of a significant proportion of the practitioners in the current study. Nonetheless, this statement is somewhat ambiguous as it is not clear whether these patients sought chiropractic care in addition to, or instead of, conventional care. In addition, more than half of the respondents recognised the need for patients in wheelchairs to use multiple therapies to effectively manage their condition, suggesting that the chiropractor's perceived role is to co-manage these patients with their GP or other healthcare professional. Of note, such an integrated approach has been cited as the preferred model from the disabled patient's perspective [9].

### GP Awareness

GPs are the gatekeepers of healthcare for patients with disabilities [18]. However there is conflicting evidence regarding the availability of CAM via GP referral [8,19]. Schmit et al [19] found that GPs in the UK were reluctant to refer patients to CAM practitioners due to a perceived lack of scientific evidence regarding their usefulness and safety. The GPs also believed establishing a scientific base would help them determine which therapy would be most appropriate for a particular patient. These concerns are therefore likely to negatively impact on the number of wheelchair-users referred to chiropractors particularly as GPs are the most frequently accessed healthcare professional by these patients [18]. In the current study approximately half of chiropractors sampled believed the GP of the wheelchair-user was aware their patient was receiving chiropractic care. This figure may be inaccurate as it is not known whether the practitioner informed the GP themselves. Greater inter-professional communication between chiropractors and GPs are vital however, to facilitate optimal care for wheelchair-users.

### Access Issues

The first step in achieving equal access to healthcare is to recognise how disabled patients experience barriers [11]. It is therefore difficult to assess the true accessibility of the clinics in the current study as only practitioner opinion was sought. Moreover, a wheelchair-user had never presented to approximately one third of the respondents. Thus chiropractors may be unaware of potential difficulties for wheelchair-users trying to utilise their services. Accessibility is an important consideration for healthcare providers as delay in receiving care may lead to increased chronicity of new conditions, seriousness of symptoms and an overall reduction in health status. Furthermore, facilities which provide good access are likely to have high levels of patient satisfaction in terms of the provision of care [20].

The criteria for accessibility in the current survey were based on the recommendations of the Office for Disability Issues (ODI) [21]. These requirements were used as the DDA requirements were considered too broad and would therefore not give specific insight into the facilities currently available in the clinics sampled. In the current study there appeared to be a disparity between practitioner perceptions of accessibility and true accessibility. For example, clinics which had disabled parking and ramp access did not always have doorways or treatment rooms large enough to accommodate a wheelchair. Therefore the usefulness of the former is negated. Only 21 (13%) clinics had all features recommended by the ODI in the current study. However, more than half of chiropractors surveyed denied any potential barriers to the treatment of a wheelchair-user despite working in clinics with limited facilities which may prevent access.

### Access and the Law

Approximately half (51%) of the chiropractors in the current study were clinic owners and therefore responsible for the compliance of their clinic with disability law. Under part III of the DDA 1995, healthcare providers are required to take all reasonable steps to avoid physical features that would make it difficult or impossible for a person with a physical disability to access their service [5]. It is outside the scope of the current study to determine whether clinics sampled were compliant with the DDA. Approximately half of the chiropractors in this study cited impracticality of alterations as a reason not to increase the accessibility of their clinic. High costs and structural limitations have been identified as potential barriers to achieving accessibility [20]. In light of this, it would have been interesting to know what equates to impracticality in the current sample. Furthermore, approximately one third of chiropractors cited lack of funds as a primary limiting factor to improved access. Despite these limitations, chiropractors should seek to maximise ease of access in whichever ways they can to ensure they do not fall short of their legal and moral obligations to these patients.

The concept of universal accessibility is a philosophy that describes full access for all people and is realised through the elimination of barriers to access for all potential users. Furthermore, it is most useful when considering moving to, or building a new clinic [20]. As a result, it is essential that new graduates or practitioners looking to open new clinics should do so with the ODI recommendations in mind. In the current study 39 (25%) chiropractors sampled would not improve access to their clinic as it would not increase patient numbers. This does not appear to be consistent with the moral code to which the profession subscribes particularly as wheelchair-users cannot become patients of a clinic they cannot access.

### Limitations of Study

There are several limitations to this study which must be considered in the interpretation of results. Firstly, the number of responses received prevents the results being generalised to other chiropractors in the UK. However, the issues raised may well exist beyond the sample studied. The reliability and validity of the questionnaire is unknown. This requires cautious analysis of the insights gained. Chiropractors were not specifically asked to consult patient files in their reporting of results therefore responses which relied on memories spanning five years increases the likihood of inaccurate recollection of information. In addition, respondents were only asked to approximate some data in order to maximise response rate which may reduce accuracy of results. Approximately one third of respondents had been in practice for less than five years. This may affect the data as they have had less patient exposure and therefore opportunity to encounter a wheelchair-user. Subjective findings must also be interpreted with caution as these were given from a practitioner point of view while in certain instances, they related more to the personal experience of the patient.

## Conclusions

Wheelchair-users appear to be an underserved patient group in relation to chiropractic services. Improvements are needed particularly in terms of physical accessibility to care, an area in which chiropractors may be viewed as failing to fulfil their ethical obligations to such patients. Among wheelchair-users who have received treatment, chiropractic management appears to be primarily utilised for pain control in patients with physical disabilities in which mobility may be improved or maintained. More research is required however regarding the effectiveness of chiropractic treatment across a range of disabilities. It would appear that in treating these patients, an integrated approach between chiropractors and GPs may well provide optimal care to wheelchair-users.

## Competing interests

The authors declare that they have no competing interests.

## Authors' contributions

NM conceived the study and collected and analyzed the data. Both authors designed the study and contributed to and approved the final manuscript.
